# False Doctrin

**Published:** 1882-12

**Authors:** 


"False Doctrin."—"Your boy looks very bad, Mrs. Jones; what's the matter?" Mrs. Jones—"Yes, ma'am, he be very bad; an' what's more, the doctors has made him worse. I'm sure we poor people need to pray with all our hearts, 'From all false doctrin, good Lord deliver us.' I never saw its meaning afore."—*Judy.* [A good prayer for the American Institute.]

